# Phytochemicals in Pancreatic Cancer Treatment: A Machine Learning Study

**DOI:** 10.1021/acsomega.3c05861

**Published:** 2023-12-27

**Authors:** Destina Ekingen Genc, Ozlem Ozbek, Burcu Oral, Ramazan Yıldırım, Nazar Ileri Ercan

**Affiliations:** † Department of Chemical Engineering, Bogazici University, Bebek, Istanbul 34342, Turkey; ‡ Department of Chemical Engineering, Middle East Technical University, Çankaya, Ankara 06800, Turkey

## Abstract

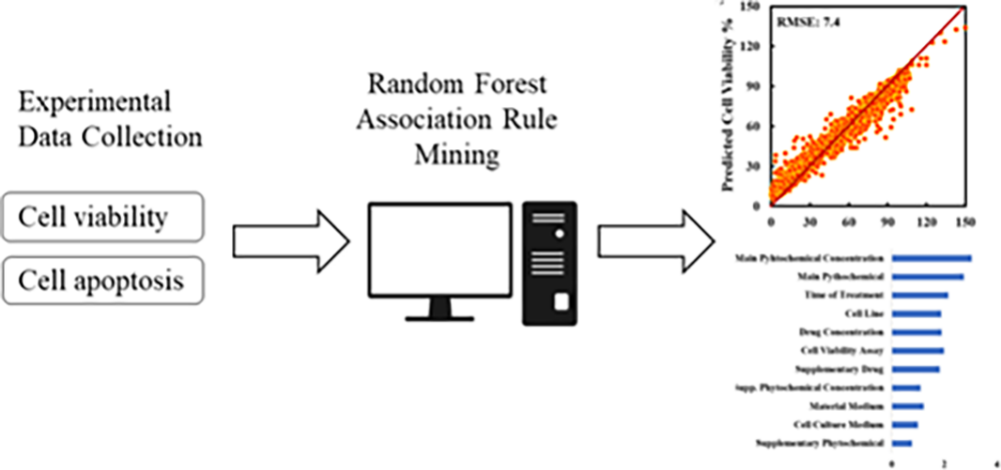

The discovery of new strategies and novel therapeutic agents is crucial to improving the current treatment methods and increasing the efficacy of cancer therapy. Phytochemicals, naturally occurring bioactive constituents derived from plants, have great potential in preventing and treating various diseases, including cancer. This study reviewed 74 literature studies published between 2006 and 2022 that conducted *in vitro* cytotoxicity and cell apoptosis analyses of the different concentrations of phytochemicals and their combinations with conventional drugs or supplementary phytochemicals on human pancreatic cell lines. From 34 plant-derived phytochemicals on 20 human pancreatic cancer cell lines, a total of 11 input and 2 output variables have been used to construct the data set that contained 2161 different instances. The machine learning approach has been implemented using random forest for regression, whereas association rule mining has been used to determine the effects of individual phytochemicals. The random forest models developed are generally good, indicating that the phytochemical type, its concentration, and the type of cell line are the most important descriptors for predicting the cell viability. However, for predicting cell apoptosis the primary phytochemical type is the most significant descriptor . Among the studied phytochemicals, catechin and indole-3-carbinol were found to be non-cytotoxic at all concentrations irrespective of the treatment time. On the other hand, berbamine and resveratrol were strongly cytotoxic with cell viabilities of less than 40% at a concentration range between 10 and 100 μM and above 100 μM, respectively, which brings them forward as potential therapeutic agents in the treatment of pancreatic cancer.

## Introduction

Pancreatic cancer is highly prevalent and one of the most fatal forms of cancer worldwide. According to the American Cancer Society Cancer Statistics Center, pancreatic cancer is estimated to become the 10th most common cancer type, and its mortality is estimated to rise to third place among the other cancer forms in 2023.^1^

Although the inheritance of pancreatic cancer constitutes approximately 10% of the cases,^2^ independent risk factors, including smoking,^2^ obesity,^3^ and alcohol intake,^4^ were also found to be influential in cancer development. Some studies demonstrated a relationship between pancreatic cancer with gender and age as well;^5^ men are more likely than women to have pancreatic cancer,^6^ and increased age positively correlates with the incidence and death rates.^7^

Radiation, chemotherapy, and immunotherapy are the alternative methods for pancreatic cancer treatment; however, the most effective way is still to undergo surgery.^8^ In chemotherapy, one of the most widely used methods, many drugs, including gemcitabine,^9^ 5-fluorouracil,^8^ capecitabine,^10^ and methotrexate,^11^ are used as therapeutic agents. Yet, these drugs might have side effects like dosage limitations and chemoresistance.^12^ Therefore, alternative approaches are required to discover new potential drugs or their combinations with traditional medications.

Phytochemicals are naturally occurring biologically active substances derived from plants.^13^ Several phytochemicals were shown to possess antitumor activities and are promising tools to enhance the efficiency of cancer treatment and lower adverse reactions.^13^ It was reported that plant-based substances and phytochemicals increase drug sensitivity against drug resistance during the treatment.^14^ Examples of plant-derived anticancer drugs include paclitaxel, docetaxel, homoharringtonine, camptothecin, vincristine, and vinblastine, which are used in the treatment of breast cancer, lung cancer, stomach cancer, prostate cancer, ovarian cancer, melanoma, neuroblastoma leukemia, thyroid cancer, and more.^14^ Various plant-derived phytochemicals such as apigenin, baicalein, crocetin, emodin, evodiamine, gallic acid, epigallocatechin gallate (EGCG), curcumin, harmine, thymoquinone, and resveratrol are found to show anticancer properties in pancreatic cancer cells.^15^ Both *in vivo* and *in vitro* studies regarding cytotoxicity analyses of these phytochemicals in a concentration and exposure time manner and their apoptotic activities on cancer cells and identification of mechanisms of action are crucial to discovering their potential therapeutic activities.

Signaling pathways also significantly impact pancreatic cancer development and its treatment. Some critical signaling pathways in pancreatic cancer include phosphoinositide 3 kinase AKT mammalian target of rapamycin (PI3K-AKTmTOR),^17^ which is related to the cell cycle; c-Jun N-terminal kinase (JNK), which is involved in cell apoptosis;^18^ Hedgehog signaling (Hh), which is linked with cell proliferation;^19^ signal transducer and activator of transcription 3 (Stat3), which is critical for tumorigenesis,^20^ and the MEK/ERK (extracellular signal-regulated kinase) pathway, which is closely related with the Notch signaling pathway, i.e., cell proliferation.^21,22^ Nuclear factor kappa-light-chain-enhancer of activated B (NF-κB) has a close relation with cell proliferation and cell death,^23^ whereas nuclear factor erythroid 2-related factor 2/NFE2L2 (Nrf2) is related to the oxidative stress response.^24^ Drugs or chemicals can regulate specific signaling pathways, and their role in the treatment could be observed.^16^ In this context, the above-mentioned signaling pathways were reported to be regulated by the exposure of specific phytochemicals.

Understanding the effects of phytochemicals on pancreatic cancer is necessary to identify potential drug variants for treatment. At this point, machine learning (ML), as a subfield of artificial intelligence, can be beneficial in overcoming experimental limitations by achieving a more comprehensive understanding of the effects of various phytochemicals under different conditions. ML is widely used to learn from the experiences hidden in large data sets; it uses statistics and some algorithms that can help to see the patterns in the data, make predictions, or develop heuristic rules to guide experimental or clinical studies in the future.^25^ Indeed, as in the other fields of science and medicine, several groups have employed machine learning algorithms to understand the effectiveness of phytochemicals on different diseases. For example, Yoo et al. predicted the drug-like properties of herbal compounds via deep learning analysis of 4507 natural compounds and 2882 approved and investigational drugs,^26^ whereas Wardani et al. performed a bioinformatic study of citrus flavonoids as chemopreventive agents in liver cancer.^27^ Similarly, Veselkov et al. analyzed a database of 7962 bioactive molecules within foods to discover cancer-beating molecules using an ML model trained by 1962 approved drugs (199 of them were anticancer drugs).^28^ Finally, Lu et al. recently used the random forest technique to understand the effectiveness of traditional Chinese medicine against acute pancreatitis.^29^

In the present study, we have reviewed 74 articles, including *in vitro* cell viability and apoptosis analyses of phytochemicals on human pancreatic cell lines, published between 2006 and 2022. From these studies, we have collected the results from 34 plant-derived phytochemicals on 20 human pancreatic cancer cell lines and investigated a total of 2160 different cases. After the preliminary analyses were performed to understand the database structure, we developed predictive random forest models and association rules to see the effects of phytochemical type and concentrations. As far as we know, the closest study to our work is the paper by Lu *et al.*;^29^ however, they used the efficiency of prescriptions prepared by traditional Chinese medicine in the treatment of acute pancreatitis, whereas our data set was constructed from scientific publications showing the effects of phytochemical type and concentration on the viability and apoptosis of pancreatic cancer. To the best of our knowledge, no such work has been published so far.

## Methods

### Data Set Construction

The data set was constructed through an extensive online research, including Google Scholar, Web of Science, and PubMed pages. Keywords of ″pancreatic cancer″, ″phytochemicals″, ″cell viability″, ″cell apoptosis″, ″combined treatment″, and ″potential drug″ were searched in studies that were published between the years 2006 and 2022. Seventy-four articles were included in the data set with 2160 data points. We identified the phytochemical type, phytochemical concentration, phytochemical exposure time, phytochemical medium, pancreatic cancer cell line, and cell culture medium as the input variables (descriptor), whereas the percent cell viability and the rate of cell apoptosis were the target variables. We also added the cell viability assay type in studies investigating cell viability, the apoptosis assay type in studies investigating cell apoptosis, and the information on drug and adjuvant phytochemicals when phytochemicals and/or drugs were used in combination therapy. Graphical data of the cell viability and cell apoptosis in the selected articles were extracted with WebPlotDigitizer 4.6.^30^ There were 2011 data points for the cell viability data set and 336 data points for the apoptosis data set collected in total. The Excel file containing the data set is given in Supporting Information S1, whereas the descriptor sets and target variables are provided in Tables S1 and S2 in Supporting Information S2.

### Model Development

All predictive models were developed by using R and Rstudio. Random forest with the package of *randomForest*(31) was used as a regression algorithm, whereas association rule mining (ARM), with the *arules*(32) package, was used to investigate the effect of individual variables on the output. Although every instance represents an independent experiment, studies involving the change of the material’s concentration were grouped under the same experiment number to prevent data leakage (i.e., experiments conducted under the same conditions using different concentrations of the same phytochemical should not be divided into training and testing sets because they are not independent).

Association rule mining correlates the descriptors with output variables and deduces a set of rules from the data set; the strength of the rules is assigned according to appearance frequency in the data set, indicated by three parameters: *support*, *confidence*, and *lift*. The meaning and significance of these parameters will be discussed in detail in the Results and Discussion section through examples. In short, the lift value is the most important measure for the strength of the rules, and the support value shows how frequently that rule is observed in the data set. A lift value higher than 1 indicates a strong correlation between the *X* and *Y* variables. ARM analysis requires categorical (non-numeric) data to find a correlation between variables; therefore, numeric values in our data sets were discretized. The concentration values were grouped as none (meaning no usage of that material, 0 μM), 0–10, 10–100, and above 100 μM. The cell viability values were grouped as cytotoxic and non-cytotoxic; values above 70% were assigned to be non-cytotoxic, whereas values below and equal to 70% were considered cytotoxic following ISO 10993-5. To distinguish the strong cytotoxicity of the material from others, we also applied ARM by dividing the data into four classes as strongly cytotoxic (cell viability equal to or below 40%), moderately cytotoxic (41–60%), weakly toxic (61–80%), and non-cytotoxic (81–100%) per ISO 10993-5. Although we tried to consider the established assumptions and discussion in the literature, all of these values were arbitrary and were selected to give a general idea about the chemicals. If needed, then these limits could be easily changed and a new set of rules could be created.

In random forest regression, the data set was split into 80–20% train–test sets according to experiments (i.e., particular experiment values were either in the train or test set), and the train set was used for the model development, whereas the test set was used for the model performance evaluation. Missing values in the input variables were filled with the mean and mode of the training set.

*k*-fold cross-validation was used for model hyperparameter tuning, which was the number of trees (ntree) and the number of features (mtry) for the random forest; they were optimized via a grid search for the random forest model. *k* was selected as 10 for the viability data set and 5 for the apoptosis data set, respectively, since the lowest RMSE values were achieved with those *k* values. The lowest validation RMSE giving the hyperparameter set was selected for model building. ntree and mtry were found to be 310 and 6 for the viability data set and 130 and 3 for the apoptosis data set.

## Results and Discussion

### Preliminary Analyses

**Figure 1:**
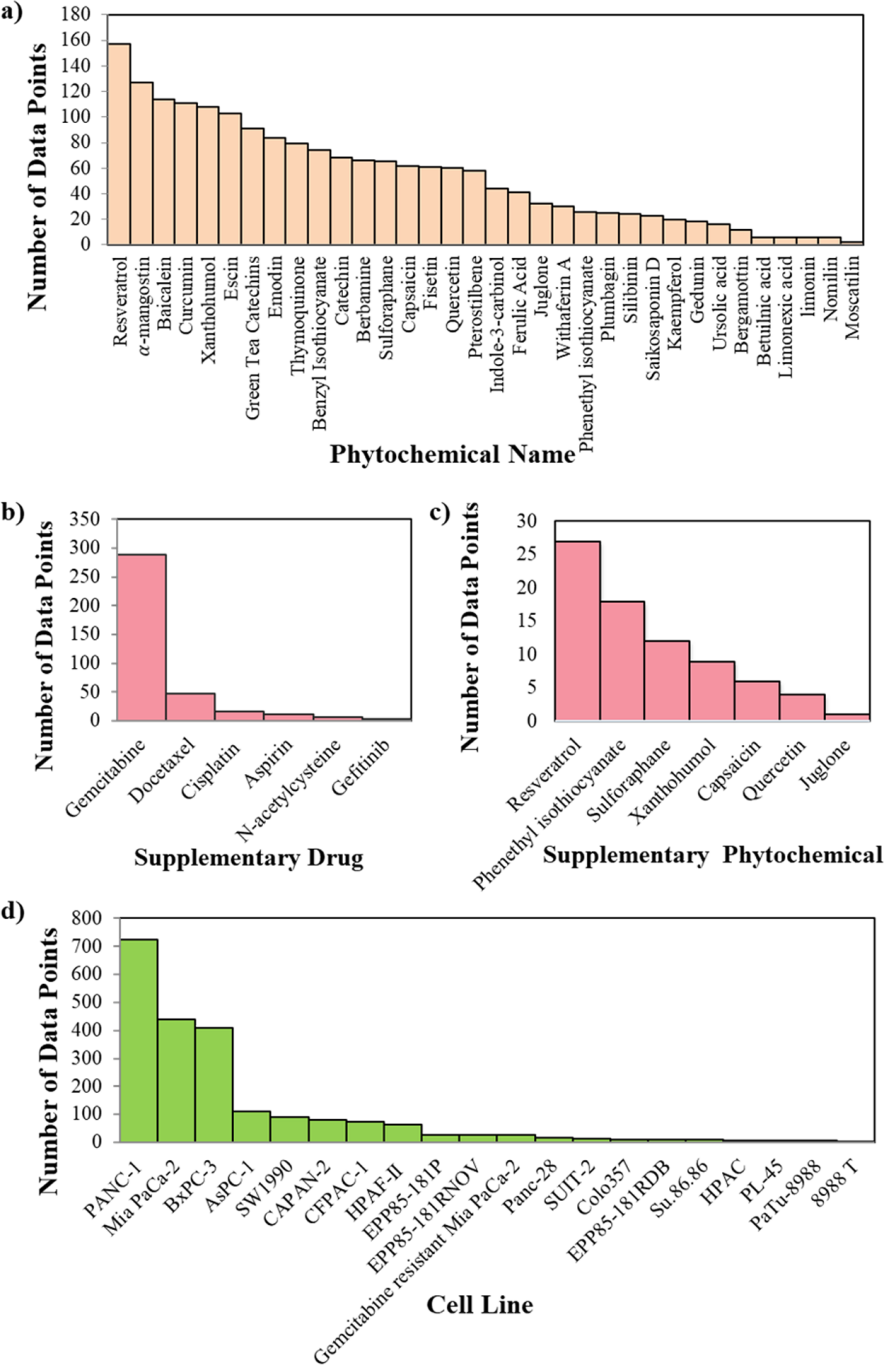
Number of data points for the (a) primary phytochemical, (b) supplementary drug, (c) supplementary phytochemical, and (d) cell line present in the data set.

For the preliminary analyses, simple descriptive statistics were used to overview the data set before starting detailed analyses via machine learning. The number of data points in the data set for the primary phytochemicals is given in Figure 1a. Resveratrol is the phytochemical that appeared with the highest frequency (157 data points) in the data set followed by α-mangostin and baicalein, which have 127 and 114 data points, respectively. Curcumin, xanthohumol, and escin also have relatively high data points (111, 108, and 103 data points, respectively).

Combining the chemical or the drug with another active substance has been a preferred method to increase the chemical’s or the pharmaceutical’s effectiveness.^33^ This also applies to our present data set where 374 cases investigated the combined effect of phytochemicals with drugs, including aspirin, cisplatin, docetaxel, gefitinib, gemcitabine, and *n*-acetylcysteine. Among these drugs, gemcitabine is the most studied, with 77% of presence in the data set (Figure 1b). Similarly, phytochemicals combined with other phytochemicals result in higher effectiveness.^34^ In this context, phytochemicals, such as capsaicin, juglone, phenethyl isothiocyanate, quercetin, resveratrol, sulforaphane, and xanthohumol, are the commonly studied supportive agents (Figure 1c). Among these, resveratrol is the most preferred supplementary phytochemical, similar to the findings of the primary phytochemicals.

Exposure of phytochemicals to 20 different human pancreatic cell lines was recorded in this study. Figure 1d shows the frequency of each recorded cell line type. The most studied cancer cell line is PANC-1, with 723 data points. This is followed by Mia PaCa-2 and BxPC-3, with 440 and 408 data points, respectively. AsPC-1 and SW1990 also have relatively high data points in the data set (i.e., 109 and 91, respectively).

Selection of an appropriate cell medium is also vital to ensure that the anticancer activity is due to the effectiveness of the phytochemical and not the lack of nutrition. Cell lines might grow in different media; however, suitable growth media should be selected to achieve optimal growth. Also, different cell viability assay types can measure the cell viability. However, these factors are secondary in the order of importance compared with the factors listed above. The distributions of cell culture media and viability assay types and further discussions on those are provided in the Supporting Information S3.

To investigate the most effective phytochemicals in human pancreatic cancer cell lines, we arbitrarily defined the following criteria: First, the cell viability percentage of the cells exposed to phytochemicals should be lower than 60%, and the cell apoptosis percentage should be higher than 30%. Based on this criteria, five phytochemicals, namely, berbamine, curcumin, escin, withaferin A, and saikosaponin D, are found to be effective against pancreatic cancer cells (cf. Figures S4 and S6). When the signaling pathways modified by these promising phytochemicals have been analyzed, four cell signaling pathways become prominent: STAT3, NF-κB, ERK-based, and caspase-3/PARP. Berbamine is effective in inhibiting the STAT3 signaling pathway, whereas escin affects the inhibition of NF-κB signaling. On the other hand, curcumin causes the activation of caspase-3/PARP and inhibition of ERK-based signaling.

### Prediction of Cell Viability

**Figure 2:**
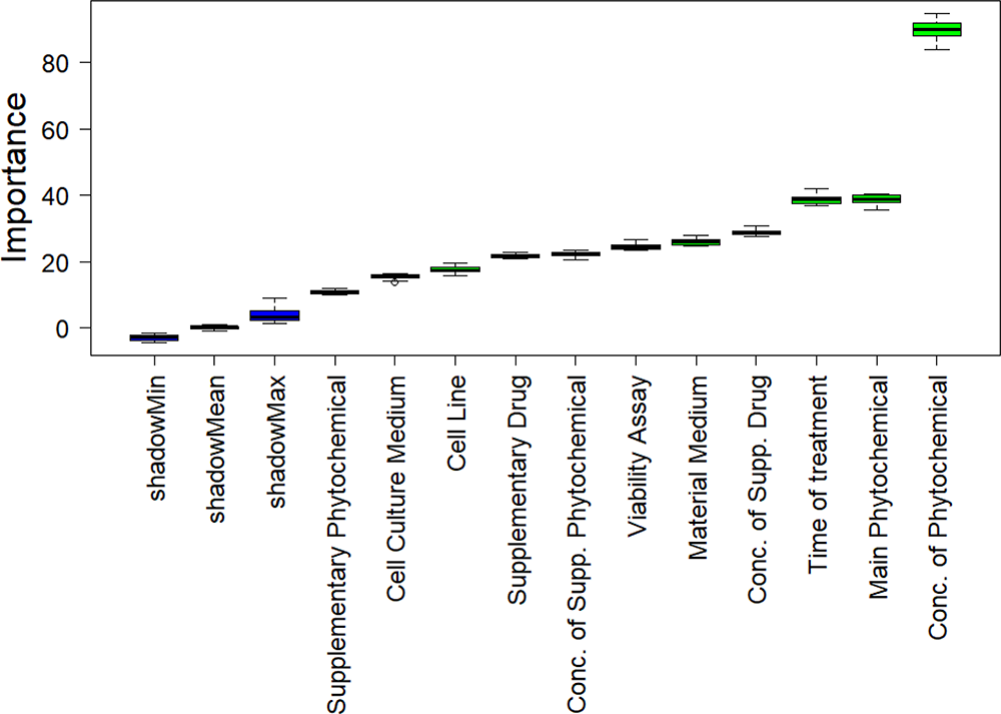
Boruta feature importance analysis for cell viability.

**Figure 3:**
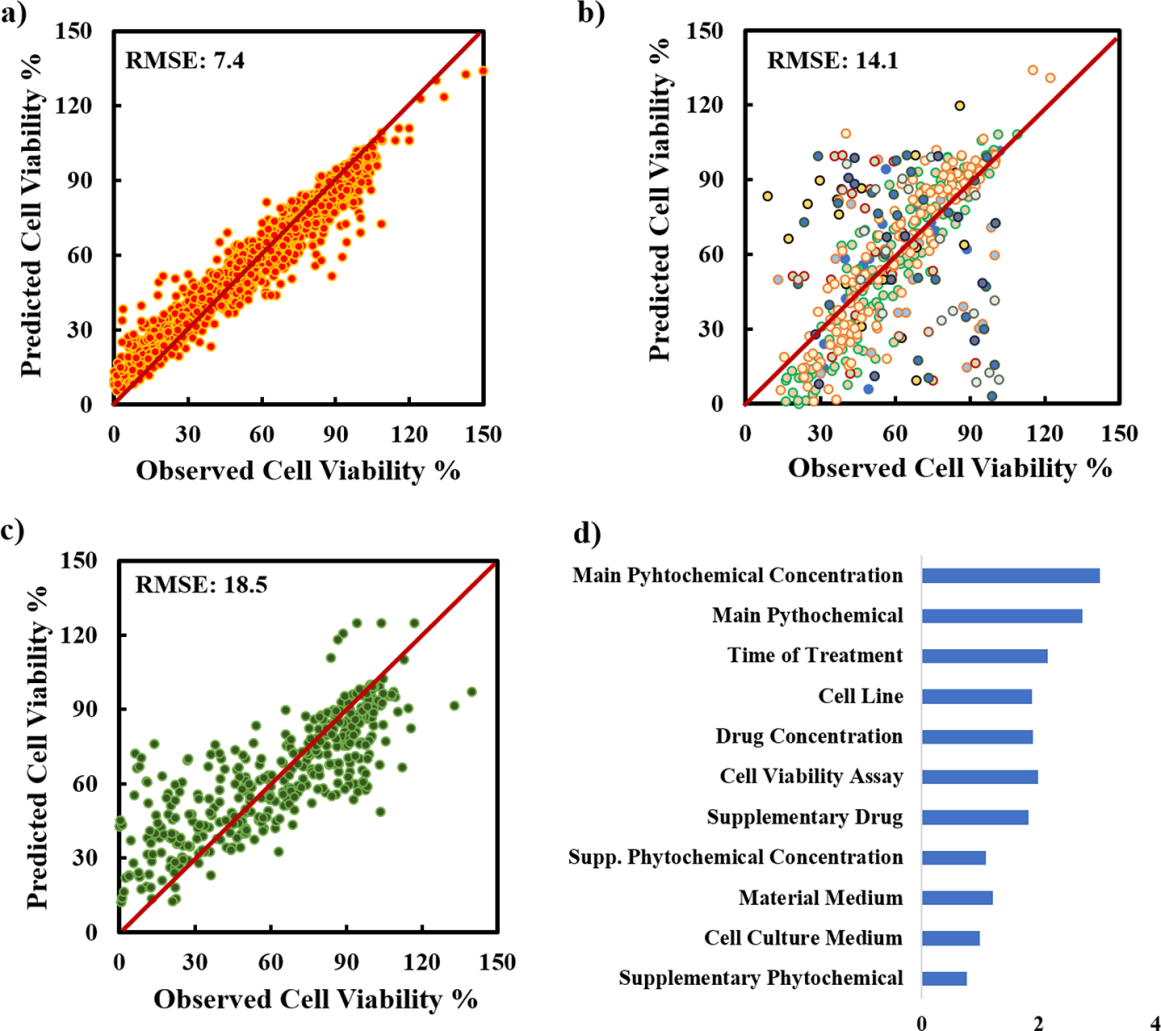
Random forest model results for the viability data set: (a) training set prediction, (b) validation set prediction, (c) testing set prediction, and (d) variable importance results.

Before developing the random forest model, a Boruta analysis was performed to identify the descriptors to be selected for model building (Figure 2); as all descriptors were found to be important, we used all of them in developing the random forest model. The predicted versus experimental cell viability plot for the cell viability data set is given in Figure 3. The prediction accuracy of the model (ntree and mtry = 310 and 6) for the training set (Figure 3a) is quite high (RMSE of 7.4). Moreover, the fitness of the validation (Figure 3b) and testing set (Figure 3c), which is a better indicator of the predictive power of the model, is also found to be satisfactory (14.1 and 18.5, respectively). Most data points are distributed well around the *x* = *y* line. Still, there is a tendency to overpredict the low-viability data (below 50%) as they are more apparent for the testing set. This attributed to the fact that the data points above 100%, which are large in number, influence the prediction more, reducing the contribution of low-viability data; different experimental error levels at high- and low-viability data or unintentional omission of descriptors that may affect the low-viability predictions more may be the other possible reasons for this result.

Tree-based models split the data according to some criteria using input variables. Each split results in two nodes: one node includes the data that follow the criterion at hand, and the other node contains the data that do not follow it. As the tree grows, each node splits further with other measures; this way, data can be generalized using some rules. The mean square error (MSE) metric assesses the significance of a variable based on the change in mean square error. When the value of a variable is randomly changed, the change in MSE shows that it determines the importance of that variable. This metric can assess the relative importance of descriptors for predictions. Figure 3d shows that the concentration of the main phytochemical, type of the main phytochemical followed by the time of treatment, and cell line are important input variables in predicting the cell viability percentage.

### Prediction of Cell Apoptosis

**Figure 4:**
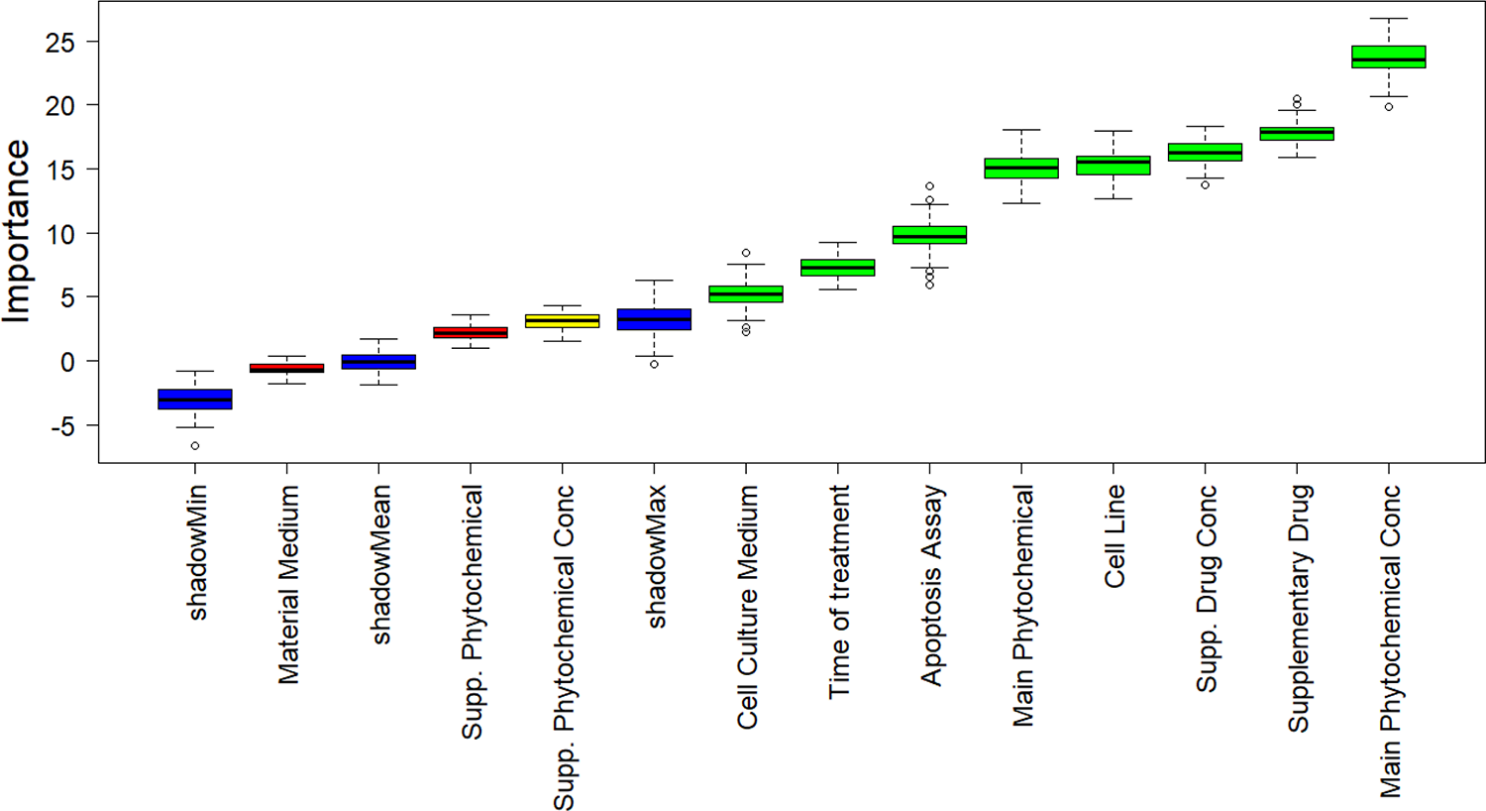
Boruta feature importance for the apoptosis data set with log-transformed output.

The performance of the random forest model was not good for the cell apoptosis data set, probably due to its relatively small size (336 data points against 2011 for cell viability (see Supporting Information)). Therefore, we transformed the output to log scale (10 based log) to see whether an order of magnitude prediction is possible (we added 1.0 to all data to eliminate zeros). Again, we started with Boruta feature analysis to select the critical variables and found that the material medium and the supplementary phytochemical type are not important for the log scale prediction of cell apoptosis (Figure 4). The most plausible explanation for this is that there are only two types of material medium, i.e., DMSO and PBS, for this data set, and the majority of the data (96%) involves DMSO. Hence, there are no sufficient number of data points to counterbalance DMSO and reliably show its effects relative to the others. Similarly, only capsaicin and sulforaphane are utilized among the supplementary phytochemical types, whereas 98% of data contain no supplementary phytochemicals. Consequently, the random forest model was developed by excluding these variables.

**Figure 5:**
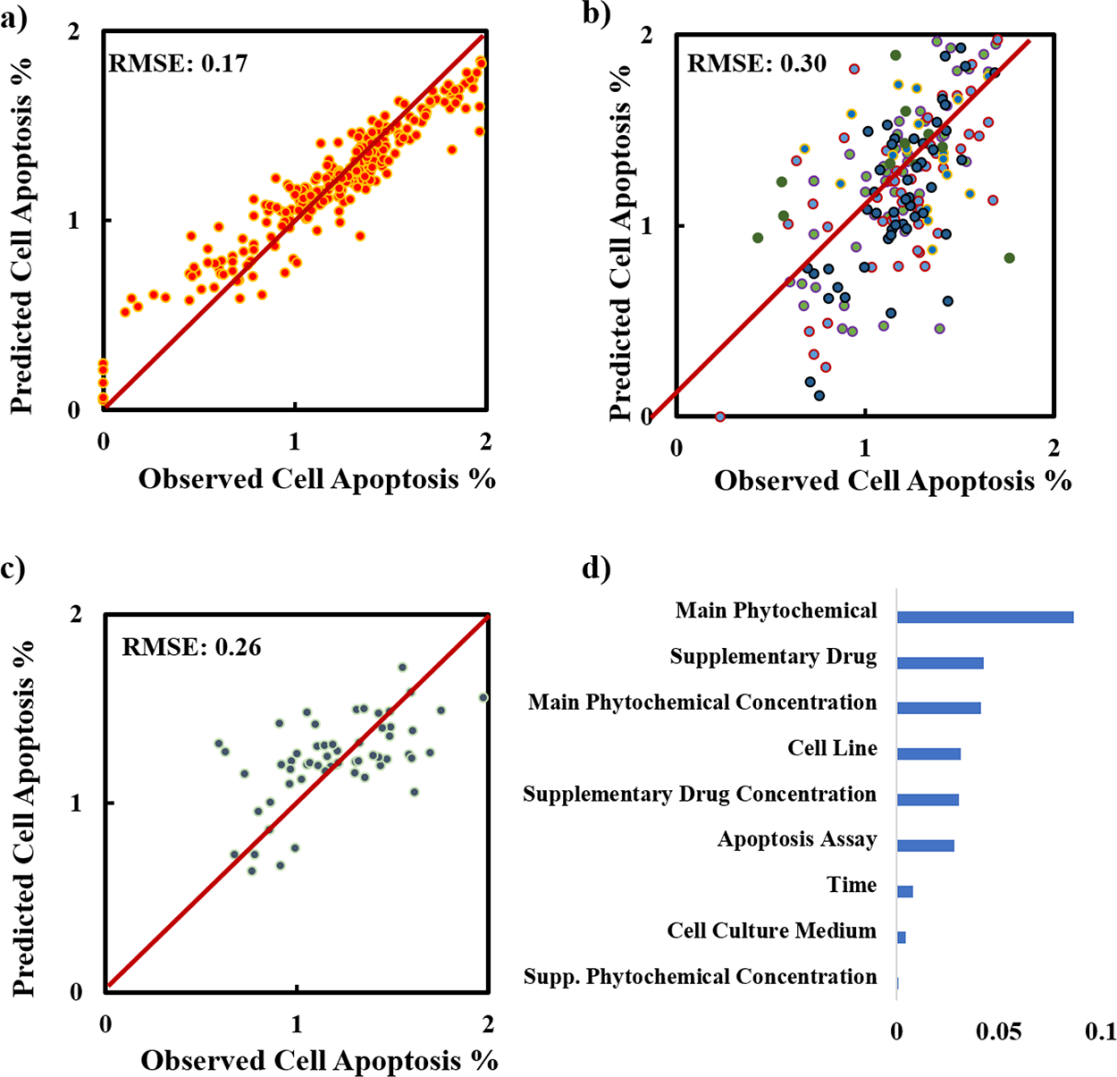
Random forest model results for the apoptosis data set: (a) train set prediction, (b) test set prediction, (c) testing set prediction, and (d) variable importance results.

The prediction of the model performance (ntree and mtry as 130 and 3) can be seen in Figure 5. Because the data number is low for apoptosis, the predictions for the test set are not as powerful as those for cell viability. The training set is accurately predicted with a training RMSE of 0.17 (Figure 5a), validation RMSE of 0.30 (Figure 5b), and testing RMSE of 0.26 (Figure 5c). When the log transformation was reversed, the RMSEs of training, validation, and testing were 10.5, 17.0, and 13.2, respectively. The variable importance plot (based on the MSE metric) (Figure 5d) suggests that the main phytochemical and its concentration, as well as supplementary drug type have the highest importance for predicting apoptosis compared to other variables. Supplementary phytochemical concentration is the least important variable in the prediction, as expected, because its significance was not strong in the Boruta analysis (shown in yellow), and supplementary phytochemical type is not in the set of descriptors used for the apoptosis prediction. It should be noted that Boruta analysis was only used to assess the descriptors’ significance in the prediction, and the strength of the variables in the prediction of apoptosis should be obtained from the variable importance plot of the best model (Figure 5d) because Boruta analysis was not performed under optimum model parameters.

### Association Rule Mining Analysis for Cell Viability

Although feature (descriptors’) importance results (Figures 4d and 5d) provide some insight into the contribution of various descriptors to the model, a more detailed analysis is needed to observe the direct effects of the individual descriptors on cell viability and apoptosis. Therefore, ARM was applied to deduce some associations for the cytotoxicity of phytochemicals using cell viability data. In contrast, the apoptosis data size was insufficient for this analysis.

The ARM results for single- (only between cell viability and one descriptor) and double- (between cell viability and two factors) factor associations are given in Tables 1 and 2, respectively (the label “main” is used for the primary phytochemical, whereas “drug” is used for the chemicals already used in the treatment). To understand the meaning of the list in the tables, we will briefly explain the parameters used in ARM in an example. In Table 1, the catechin is labeled as non-toxic (i.e., cell viability is above 70%) with support, confidence, and lift values of 0.03, 0.9, and 2, respectively. *Support* here indicates the fraction of cases that use catechin and are found to be non-toxic in all data corresponding to 61 points as counted in the table (2011 × 0.03 = 61, as the actual value of support is 0.0303); *support* reflects the fraction or number of cases to support the rules stated in that row in the table. *Confidence* is 61/68 = 0.9, indicating that 90% of catechin cases are non-cytotoxic as the indicator of the reliability of the rule stated. Then, *lift* means the fraction of non-toxic cases of catechin against the fraction of non-toxic instances in the entire data set (0.9/0.45); we can express this as the probability of having non-cytotoxic cases involving catechin 2 times higher than the probability of finding non-cytotoxic points in the entire data set as a strong indicator of the non-cytotoxicity of catechin.

**Table 1:** Single-Factor Associations for Cytotoxicity

variables	viability	support	confidence	coverage	lift	count
main phyto. = catechin	non-cytotoxic	0.03	0.90	0.04	2	61
main phyto. = indole-3-carbinol	non-cytotoxic	0.02	0.82	0.02	1.8	36
drug = docetaxel	cytotoxic	0.01	0.89	0.01	1.6	24

**Table 2:** Double-Factor Associations for Cytotoxicity

variables	viability	support	confidence	coverage	lift	count
main phyto. = berbamine, conc. phyto = 10–100 μM	cytotoxic	0.01	1	0.01	1.8	23
main phyto. = capsaicin, conc. phyto = above 100 μM	cytotoxic	0.02	1	0.02	1.8	35
main phyto. = sulforaphane, conc. phyto = 10–100 μM	cytotoxic	0.02	1	0.02	1.8	32
main phyto. = benzyl isothiocyanate, conc. phyto = 10–100 μM	cytotoxic	0.02	0.97	0.02	1.8	34
main phyto. = baicalein, conc. drug = 0–10 μM	cytotoxic	0.02	0.94	0.02	1.7	34
main phyto. = baicalein, drug = gemcitabine	cytotoxic	0.01	0.93	0.01	1.7	25
main phyto. = resveratrol, conc. phyto = above 100 μM	cytotoxic	0.02	0.91	0.02	1.7	40
drug = docetaxel, time of treatment = 48 h	cytotoxic	0.01	0.89	0.01	1.6	24
drug = docetaxel, conc. drug = 0–10 μM	cytotoxic	0.01	0.89	0.01	1.6	24
main phyto. = curcumin, conc. phyto = 10–100 μM	cytotoxic	0.03	0.89	0.03	1.6	54
drug = gemcitabine, conc. drug = 10–100 μM	cytotoxic	0.01	0.88	0.01	1.6	23
main phyto. = curcumin, time of treatment = 72 h	cytotoxic	0.02	0.86	0.02	1.6	31
main phyto. = α-mangostin, conc. phyto = 10–100 μM	cytotoxic	0.02	0.85	0.02	1.6	35
main phyto. = thymoquinone, conc. phyto = 10–100 μM	cytotoxic	0.02	0.85	0.03	1.6	40
main phyto. = escin, conc. phyto = 10–100 μM	cytotoxic	0.03	0.84	0.03	1.5	51
main phyto. = resveratrol, time of treatment = 48 h	cytotoxic	0.03	0.82	0.04	1.5	56
main phyto. = baicalein, conc. phyto = 10–100 μM	cytotoxic	0.02	0.81	0.03	1.5	42
main phyto. = benzyl isothiocyanate, time of treatment = 24 h	cytotoxic	0.02	0.81	0.02	1.5	34
main phyto. = capsaicin, time of treatment = 48 h	cytotoxic	0.01	0.8	0.02	1.5	24
drug = gemcitabine, time of treatment = 48 h	cytotoxic	0.06	0.81	0.07	1.5	111
main phyto. = catechin, conc. phyto = 0–10 μM	non-cytotoxic	0.01	1	0.01	2.2	20
main phyto. = indole3carbinol, conc. phyto = 0–10 μM	non-cytotoxic	0.01	1	0.01	2.2	27
main phyto. = catechin, conc. phyto = 10–100 μM	non-cytotoxic	0.02	0.95	0.02	2.1	38
main phyto. = xanthohumol, conc. phyto = 0–10 μM	non-cytotoxic	0.01	0.93	0.02	2	27
main phyto. = catechin, supp = none	non-cytotoxic	0.03	0.9	0.04	2	61
main phyto. = catechin, drug = none	non-cytotoxic	0.03	0.9	0.04	2	61
main phyto. = catechin, time of treatment = 48 h	non-cytotoxic	0.01	0.89	0.02	2	25
main phyto. = catechin, time of treatment = 24 h	non-cytotoxic	0.01	0.89	0.02	2	25
main phyto. = indole-3-carbinol, time of treatment = 24 h	non-cytotoxic	0.02	0.82	0.02	1.8	36

In addition to catechin, the other meaningful one-factor associations are for the phytochemical indole-3-carbinol (non-cytotoxic) and drug docetaxel (cytotoxic); single-factor associations were insufficient to generalize the other phytochemicals. This was not surprising because we usually need some additional conditions to decide. For example, we typically need to know the concentration as a second criterion to determine the toxicity. Indeed, we studied two-way associations as indicated in Table 2, and in most cases, the second criterion (in addition to phytochemical) is the concentration or the treatment period, as expected.

The non-cytotoxicity behavior of catechin and indole-3-carbinol does not change with the concentration or treatment time, as seen in Table 2. Xanthohumol phytochemical with 0–10 μM concentration also shows non-cytotoxicity. Benzyl isothiocyanate, berbamine, capsaicin, sulforaphane, baicalein, resveratrol, α-mangostin, curcumin, thymoquinone, and escin show cytotoxicity under some conditions. For example, benzyl isothiocyanate shows cytotoxicity with a concentration range of 10–100 μM and with a time of treatment of 24 h, but the probability of achieving cytotoxicity is higher for 10–100 μM concentration (lift: 1.8) compared to 24 h of treatment (lift: 1.5). However, when these two conditions are combined, i.e., benzyl isothiocyanate of 10–100 μM was applied for 24 h, the lift of cytotoxicity is 1.8 (Table 3). The cytotoxicity of α-mangostin also changes with concentration. For example, when the time of treatment was 24 h and the concentration range was 0 and 10 μM, α-mangostin is found to be non-cytotoxic, whereas increasing α-mangostin concentration to 10–100 μM results in being cytotoxic. The other entries were examined in a similar manner.

**Table 3:** Double-Factor Associations for Strong Cytotoxicity

variables	viability	support	lift	count
main phyto. = berbamine, conc. phyto = 10–100 μM	strong cytotoxicity	0.01	3.2	20
main phyto. = resveratrol, conc. phyto = above 100 μM	strong cytotoxicity	0.02	3	36
main phyto. = catechin, conc. phyto = 0–10 μM	non-cytotoxicity	0.01	2.8	20
main phyto. = indole3carbinol, conc. phyto = 0–10 μM	non-cytotoxicity	0.01	2.8	27
main phyto. = xanthohumol, conc. phyto = 0–10 μM	non-cytotoxicity	0.01	2.4	25
main phyto. = catechin, time of treatment = 48 h	non-cytotoxicity	0.01	2.4	24

The results in Table 2 indicate that the phytochemical type, concentration, and treatment period determine the cytotoxicity together (there may also be some other important factors); hence, we decided to test multifactor associations, starting with three factors as well. However, no valuable information could be extracted, probably because the number of cases was insufficient for that. Additionally, we repeated the double-factor association with stronger restrictions; we labeled the data as *strongly cytotoxic* (viability lower than 40%), *moderately cytotoxic* (cell viability between 41 and 60%), *weakly cytotoxic* (61–80%), and *non-cytotoxic* (81–100%). We checked the list for strong toxicity and obtained the results in Table 3. As can be seen clearly, only a few entries in the table showed that resveratrol and berbamine are the phytochemicals with high effectiveness in reducing cell viability.

## Conclusions

In the present study, results of the previously published *in vitro* studies focused on the cytotoxic and apoptotic effects of phytochemicals on human pancreatic cell lines have been investigated by machine learning analysis. The machine learning approach has been implemented using random forest for regression, whereas association rule mining has been used to determine the effects of individual phytochemicals. The developed random forest models are generally good, indicating that the phytochemical type, its concentration, and the type of cell line are the most important descriptors for predicting cell viability. On the other hand, the primary phytochemical type has the highest importance for predicting apoptosis. Berbamine, capsaicin, sulforaphane, benzyl-isothiocyanate, baicalein, resveratrol, curcumin, α-mangostin, thymoquinone, and escin were found to be toxic at a concentration range between 10 and 100 μM or above, whereas catechin and indole-3-carbinol were mainly non-cytotoxic at all studied concentrations. Among the cytotoxic phytochemicals, berbamine and resveratrol were found to be strongly cytotoxic at a concentration range between 10 and 100 μM and above 100 μM, respectively, which brings them forward as potential therapeutic agents in the treatment of pancreatic cancer.

In summary, phytochemicals are naturally derived materials that can be effective against pancreatic cancer. Machine learning approaches can be useful in identifying the most effective materials among phytochemicals whose efficacy has already been proven by experiments. This study highlights effective phytochemicals based on previous *in vitro* studies. In the search for alternative new drugs, the current results can form a basis and be extended further by *in vitro* studies, where phytochemicals are used in combination with nanocarriers and *in vivo* studies.

## Supporting Information Available

Variables and their ranges/categories for the viability and apoptosis data sets (Tables S1 and S2); distribution of different media among pancreatic cancer cell lines (Figure S1); distribution of cell viability assay type (Figure S2); average cell viability for the most studied phytochemical types (Figure S3); average cell viability percentages versus phytochemical type (Figure S4); phytochemical vs average cell apoptosis (Figures S5 and S6); cell signaling pathways related to main phytochemicals (Figure S7); and apoptosis model with all variables without log transformation (Figure S8) (PDF)
Excel file containing the data set (XLSX)
