# Supplementary material for: Phytochemicals in Pancreatic Cancer Treatment: A Machine Learning Study
Source: ACS Omega. 2023 Dec 27;9(1):413–21. doi: 10.1021/acsomega.3c05861 (PMC10785644; doi:10.1021/acsomega.3c05861)
Supplement: Supplementary file 1 — ao3c05861_si_001.pdf [file ao3c05861_si_001.pdf]

Supplementary Material for

Phytochemicals in pancreatic cancer treatment: A machine learning study.

Destina Ekingen Genc[a‡], Ozlem Ozbek[a‡], Burcu Oral[a‡], Ramazan Yıldırım[a,], Nazar Ileri Ercan[b,*]*

[a]Department of Chemical Engineering, Bogazici University, Bebek, Istanbul, Turkey

[b]Department of Chemical Engineering, Middle East Technical University, Çankaya, Ankara

S1. The Dataset

Datasheet.xlsx

S2. Dataset Details

Table S1. Variables and their ranges/categories for viability dataset.

Variable	Range/Category
Main Pythochemical	α-mangostin, Baicalein, Benzyl Isothiocyanate, Berbamine, Betuilnic acid, Capsaicin, Catechin, Curcumin, Emodin, Escin, Ferulic Acid, Fisetin, Gedunin, Green Tea Catechins, Indole3carbinol, Juglone, Kaempferol, Limonexic acid, Limonin, Moscatilin, Nomilin, None, Phenethyl Isothiocyanate, plumbagin, Plumbagin, Pterostilbene, Quercetin, Resveratrol, Saikosaponin D, Silibinin, Sulforaphane, Thymoquinone, Ursolic acid, Withaferin A, Xanthohumol
Concentration of Material (uM)	0-200000

Supplementary Drug	Aspirin, Cisplatin , Docetaxel, Gemcitabine, *none*
Supplementary Drug Concentration (uM)	0-1000
Supplementary Phytochemical	Capsaicin, Juglone, Phenethyl isothiocyanate, Quercetin, Resveratrol, Sulforaphane, Xanthohumol, *none*
Supplementary Phytochemical Concentration (uM)	0-200000
Material Medium	Carboxymethyl cellulose sodium salt solution, DMSO, Ethanol, PBS
Cell Line	8988 T, AsPC-1, BxPC-3, CAPAN-2, CFPAC-1, Colo357, EPP85-181P, EPP85-181RDB, EPP85-181RNOV, Gemcitabine resistant Mia PaCa-2, HPAC, HPAF-II , Mia PaCa-2, PANC-1, Panc-28, PaTu-8988, PL-45, Su.86.86, SUIT-2, SW1990
Cell Culture Medium	DMEM, IMDM, Leibovitzs (L15) medium, McCoy's 5A medium, RPMI 1640
Time of Treatment (h)	0-96
Cell Viability Assay	CCK8, CTG, MTS, MTT, Presto Blue assay, Sulforhodamine B, Trypan Blue, WST8, XTT
Cell Viability (% of control)	0-152.36

Table S2. Variables and their ranges/categories for apoptosis dataset.

Variable	Range/Category
Main Phytochemical	α-mangostin, Baicalein, Benzyl Isothiocyanate, Berbamine, Bergamottin, Betuilnic acid, Capsaicin, Curcumin, Emodin, Escin, Fisetin, Gedunin, Juglone, Kaempferol, Limonexic acid, Limonin, Nomilin, None, , Plumbagin, Quercetin, Resveratrol, Saikosaponin D, Silibinin, Sulforaphane, Thymoquinone, Ursolic acid, Withaferin A, Xanthohumol
Main Phytochemical Concentration (uM)	0-250
Supplementary Drug	Aspirin, Docetaxel, Gefitinib, Gemcitabine, Nacetylcysteine, *none*
Supplementary Drug Concentration (uM)	0-10000
Supplementary Phytochemical	Capsaicin, Sulforaphane, *none*
Supp. Phytochemical Concentration (uM)	0-197
Material Medium	DMSO, PBS

Cell Line	8988 T, AsPC-1, BxPC-3, CAPAN-2, CFPAC-1, Gemcitabine resistant Mia PaCa-2, HPAC, Mia PaCa-2, PANC-1, Panc-28, SW1990
Cell Culture Medium	DMEM, McCoy's 5A medium, RPMI 1640
Time of treatment (h)	0-72
Apoptosis Assay Type	Annexin V/7-AAD staining, Annexin VEGF, Annexin VFITC, Annexin VFITC+ PI doublestaining, DAPI staining assay, Hoechst 33258 staining, TUNEL assay
Cell Apoptosis (% of total cells)	0-93.6

S3. Cell Medium, Viability Assay, Viability and Apoptosis Distributions

The cell culture medium is analyzed for 20 human pancreatic cell lines, and five culture media are recorded. The growth mediums including Dulbecco's Modified Eagle's Medium (DMEM), Roswell Park Memorial Institute 1640 Medium (RPMI-1640), Leibovitz's (L-15) medium, McCoy's 5A medium, and Iscove's Modified Dulbecco's Medium (IMDM) are present in our dataset. Figure S1 shows the count of data points for DMEM, L-15, and RPMI-1640 mediums for the cell lines used. IMDM was solely used for CFPAC-1 cell line, and similarly, McCoy's 5A medium was used only for CAPAN-2 cell line. Thus, their graphs were not shown in Figure S1. DMEM is the most preferred cell culture medium for PANC-1 cell line, and RPMI-1640 is the most preferred for BxPC-3 cell line.

Different cell viability assay types can measure cell viability. Figure S2 shows the cell viability assay types distribution among the investigated cases in this study. MTT (3-[4,5-dimethylthiazol-2-yl]-2,5 diphenyl tetrazolium bromide) assay is the most prominent assay type. With 2071 data points, it constitutes 52% of the total cases. The second most used assay type within this study is CCK-8 (Cell Counting Kit-8) which includes 17% of the cases. This is followed by MTS (3-(4,5-dimethylthiazol-2-yl)-5-(3-carboxymethoxyphenyl)-2-(4-sulfophenyl)-2H-tetrazolium) assay, which constitutes 15% of the cases.

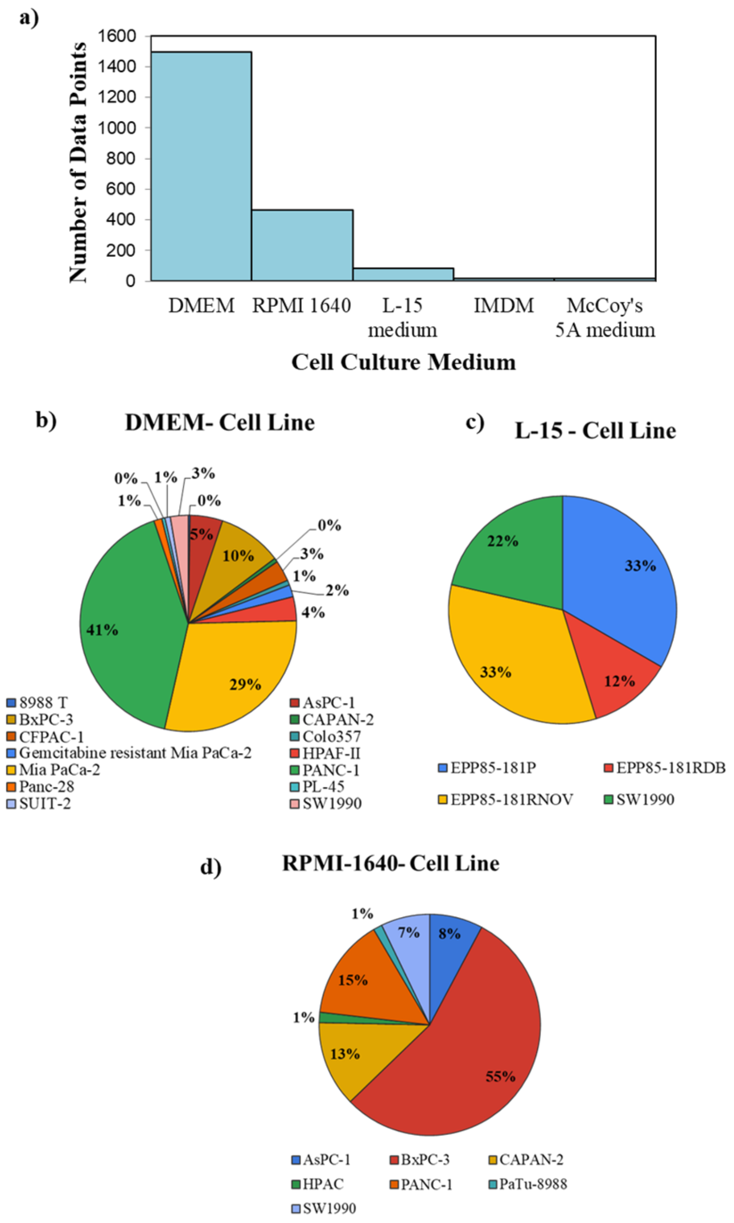

Figure S1. The distribution of a)cell culture medium among all cells, and the distribution of b) DMEM c) L-15 Medium, d) RPMI-1640 cell culture mediums among different pancreatic cell lines.

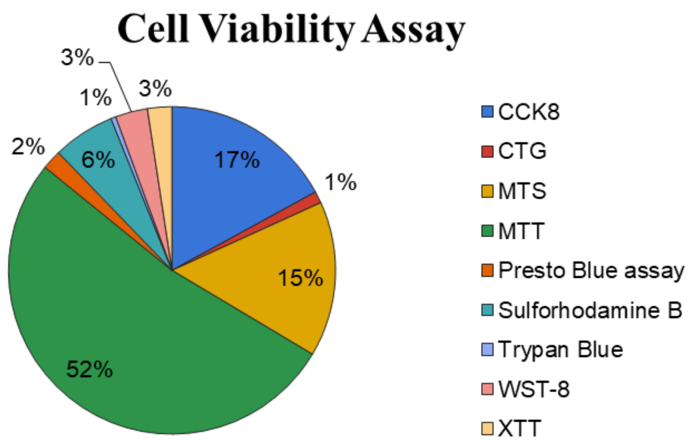

Figure S2. Distribution of cell viability assay type.

High cytotoxicity and low viability of cancer cells are necessary for effective treatment solutions. Hence, for each phytochemical type, the average viability percentage was calculated using various concentrations of the specific phytochemical irrespective of the cell line type. After applying this procedure to each of the 34 phytochemicals, the overall average cell viability percentage is found as 63.7%. The average cell viability percentages for the most studied six phytochemicals, i.e., baicalein, curcumin, escin, resveratrol, xanthohumol, and α-mangostin, are given in Figure S3. Among these, baicalein, curcumin, escin, and resveratrol resulted in lower viability percentages than the overall average cell viability, with values of 53.4%, 51.4%, 52.9%, and 56.8%, respectively. α-mangostin has an average viability percentage of 63.6% which is close to the overall average. The average viability percentage of the cells exposed to xanthohumol is 67.4% which is relatively higher than the overall average. Consequently, lower viability percentages obtained with baicalein, curcumin, escin, and resveratrol present promising results for potential treatment options in human pancreatic cancer. For all phytochemicals with average viability results that are lower than the overall average viability are given in Figure S4.

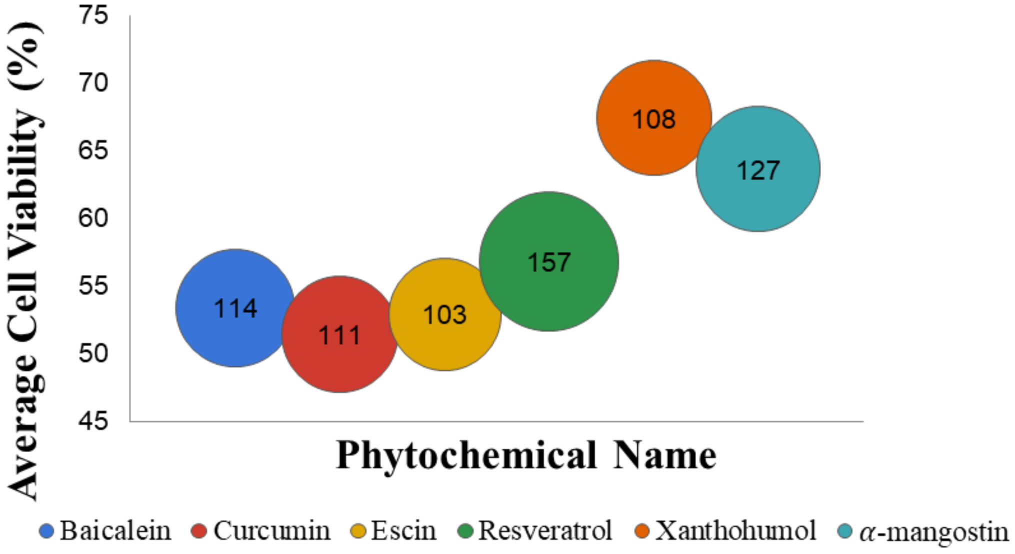

Figure S3. Average cell viability (%) for the most studied Phytochemical types baicalein, curcumin, escin, resveratrol, xanthohumol, α-mangostin.

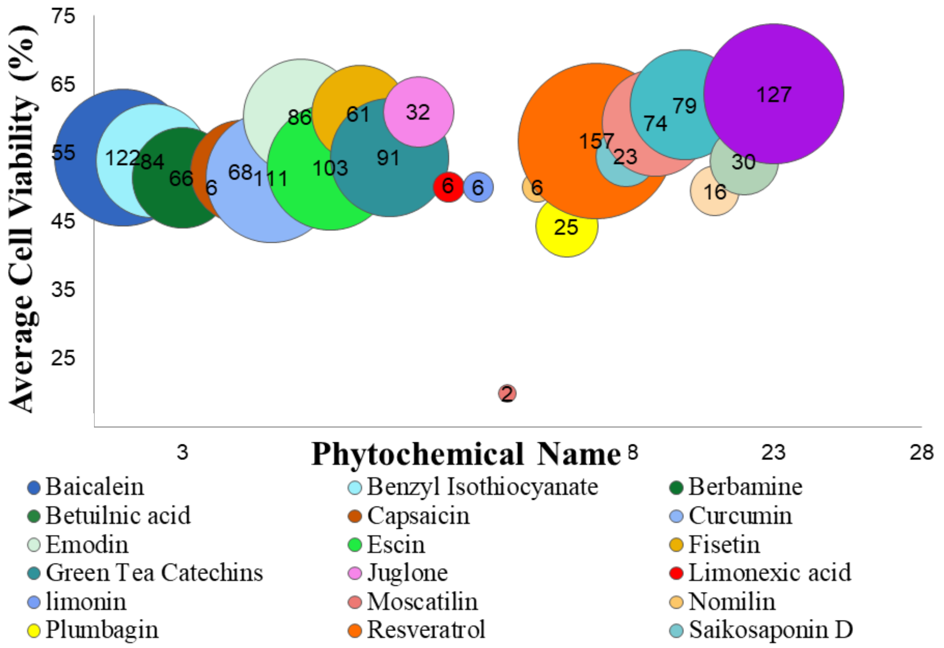

Figure S4. Average cell viability percentages versus phytochemical type graph with lower viability percentages compared to the average value.

To interpret the effectiveness of the phytochemicals, it is essential to understand their apoptotic behavior (i.e., programmed cell death) in cancer cells. To study apoptosis, four different assay types were used, including Annexin V, DAPI staining assay, Hoechst 33258 staining, and TUNEL assay. Annexin V can be used with 7-amino-actinomycin, green fluorescence protein (EGF P), or propidium iodide. Measurements were completed by flow cytometry analysis. In most of the studies, Annexin V-FITC+ PI double-staining was selected for analyzing the apoptotic behavior of the cells against the phytochemicals. Experimental studies are expected to present high apoptosis percentages for highly effective phytochemicals. The overall average percentage of cancer cell apoptosis was found to be approximately 21.45% from the dataset collected. In Figure S5, the average cancer cell apoptosis percentages are shown for the most mentioned phytochemical types. Resveratrol and α-mangostin have the highest number of apoptosis data points according to the size of the bubbles in the graph. However, α-mangostin is more effective in apoptotic behavior due to its higher % average cell apoptosis values.

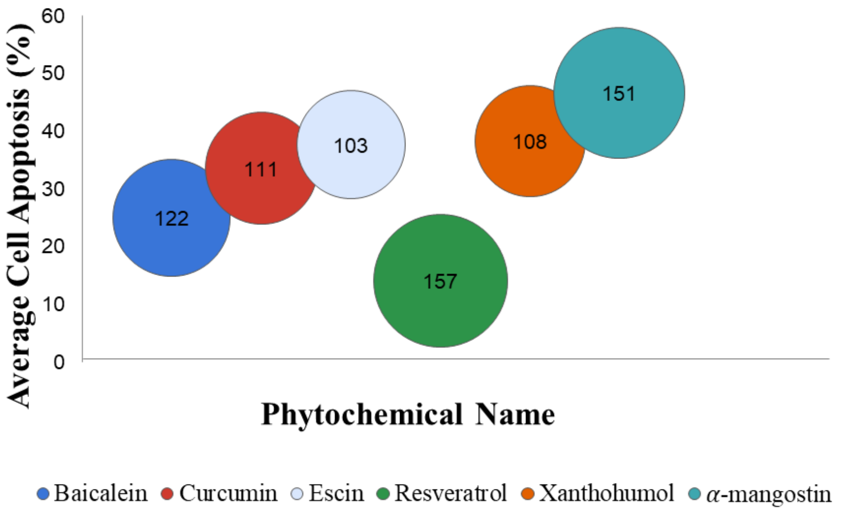

Figure S5. Phytochemical vs Average Cell Apoptosis (%) based on the number of data points.

The number of data points in the dataset and the apoptotic percentage value are essential to clearly interpret the results. Figure S6 presents the phytochemicals with apoptotic percentage values greater than or equal to the overall average apoptosis percentage, 21.45%. Berbamine, baicalein, bergamottin, capsaicin, curcumin, escin, fisetin, juglone are some of the

phytochemicals with average apoptotic percentage values that are lower than or equal to the overall average apoptosis percentage.

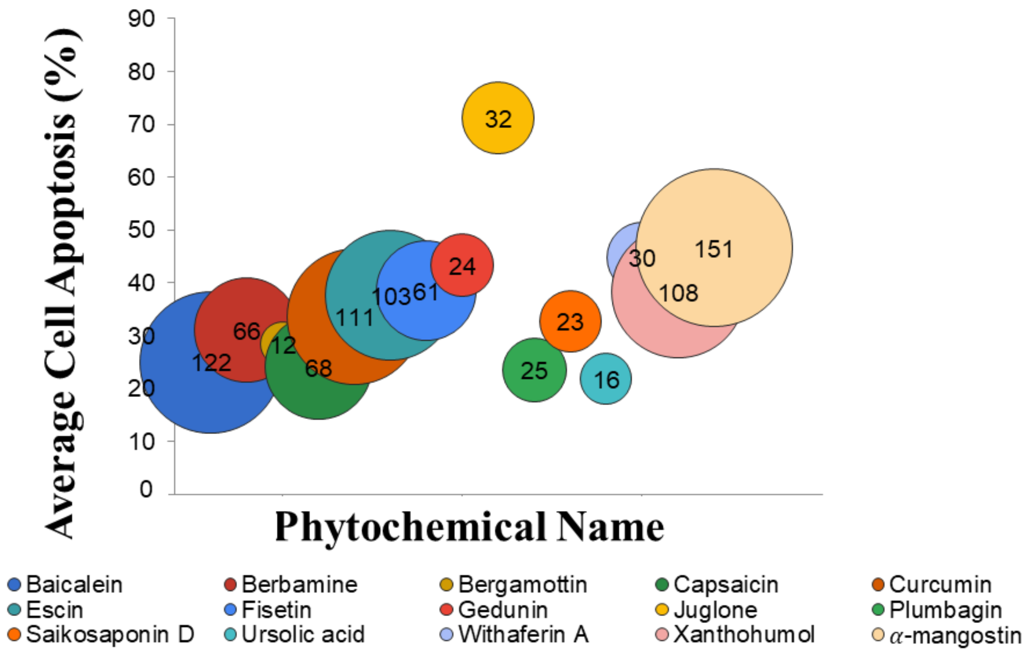

Figure S6. Phytochemical vs Average Cell Apoptosis (%) graph based on the high average apoptosis (%) values.

When signaling pathways are analyzed with respect to each phytochemical, inhibition of several signaling pathways and activation of Nrf2 and caspase-3/PARP are observed. Results are presented in Figure S7. In our dataset, inhibition of NF-kB activity is the most reported pathway (22% of reported pathways), and emodin, escin, α-mangostin, juglone, thymoquinone and fisetin causes inhibition of the NF-kB activity in the cells. Inhibition of Akt, PI3K/Akt, PI3K/Akt/mTor, and Akt/mTor pathways were grouped as Inhibition of PI3K/Akt (mTor) in Figure S7, and this pathway group is the second most reported pathway (18% of reported pathways). α-mangostin, thymoquinone (PI3K/Akt/mTor), capsaicin, kaempferol (Akt/mTor), baicalein, green tea catechins (Akt) and pterostilbene are responsible for PI3K/Akt inhibition. Inhibiton of STAT3 pathway is the third most common pathway in our dataset (16%), and α-mangostin, berbamine, quercetin, benzyl isothiocyanate, xanthohumol are reported to inhibit the STAT3 pathway. Activation of Nrf2 (11% of reported pathways) is another common pathway, and Xanthohumol, Indole3carbinol, Phenethyl Isothiocyanate, and Resveratrol activates Nrf2.

Inhibition of ERK-based signaling pathways (11% of reported pathways) consists of inhibition of ERK, ERK1/2, ERK-MYC, and MEK/ERK signaling pathways. α-mangostin, fisetin (ERK-MYC), baicalein (MEK/ERK), resveratrol and curcumin (ERK1/2) were reported to inhibit ERK-based signaling.

The most frequently studied phytochemicals are α-mangostin, baicalein, curcumin, escin, resveratrol, and xanthohumol. When their pathways are analyzed in detail, it is seen that α-mangostin can inhibit many pathways, and the distribution of the data numbers are similar. Baicalein mainly activates caspase-3/PARP pathway but also inhibits MEK/ERK and PI3K/Akt pathways. Curcumin also mainly activates caspase-3/PARP pathway, but some studies reported its contribution to the inhibition of ERK 1/2 as well (this happened only when Sulforaphane and aspirin were used as supplementary phytochemicals and drugs, respectively). Escin inhibits NF-kB activity only. Resveratrol was reported to activate Nrf2 and inhibit ERK and sonic hedgehog signaling pathways. Lastly, Xanthohumol mainly causes inhibition of STAT3, which is followed by activation of Nrf2 and inhibition of Notch1.

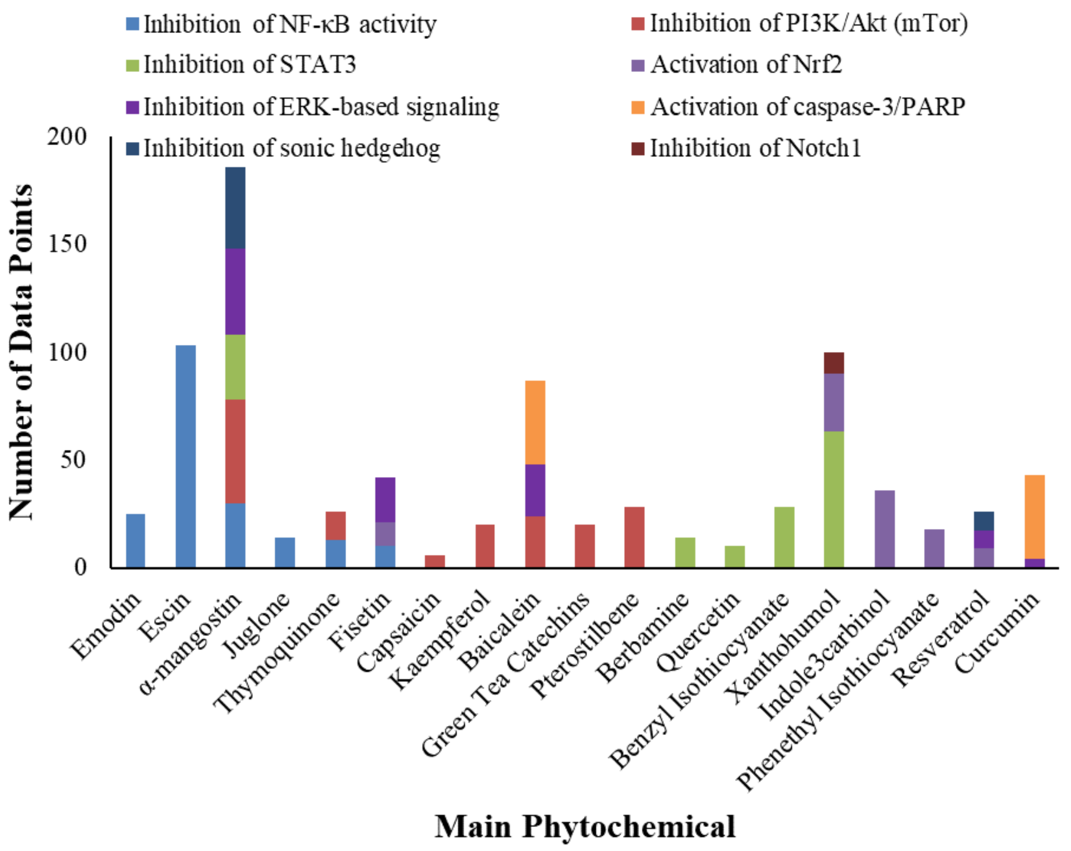

Figure S7. Cell Signaling pathways related to main phytochemicals reported in the dataset.

S4. Regression Model

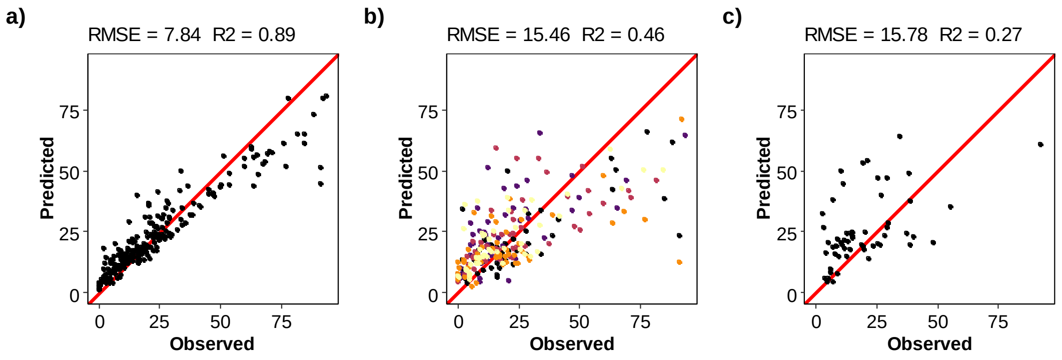

Figure S8. Apoptosis model with all variables without log transformation a) train set b) validation set c) test set results.
